# Emerging roles of dysregulated adenosine homeostasis in brain disorders with a specific focus on neurodegenerative diseases

**DOI:** 10.1186/s12929-021-00766-y

**Published:** 2021-10-11

**Authors:** Ching-Pang Chang, Kuo-Chen Wu, Chien-Yu Lin, Yijuang Chern

**Affiliations:** 1 Institute of Biomedical Sciences, Academia Sinica, Nankang, Taipei, 115 Taiwan; 2 Biomedical Translation Research Center, Academia Sinica, Taipei, Taiwan; 3 School of Pharmacy, National Taiwan University, Taipei, Taiwan

**Keywords:** Adenosine, ATP, ENTs, Mitochondria, Therapeutic treatment, Alzheimer’s disease, Huntington’s disease, Parkinson’s disease, Amyotrophic lateral sclerosis, Neuroinflammation

## Abstract

In modern societies, with an increase in the older population, age-related neurodegenerative diseases have progressively become greater socioeconomic burdens. To date, despite the tremendous effort devoted to understanding neurodegenerative diseases in recent decades, treatment to delay disease progression is largely ineffective and is in urgent demand. The development of new strategies targeting these pathological features is a timely topic. It is important to note that most degenerative diseases are associated with the accumulation of specific misfolded proteins, which is facilitated by several common features of neurodegenerative diseases (including poor energy homeostasis and mitochondrial dysfunction). Adenosine is a purine nucleoside and neuromodulator in the brain. It is also an essential component of energy production pathways, cellular metabolism, and gene regulation in brain cells. The levels of intracellular and extracellular adenosine are thus tightly controlled by a handful of proteins (including adenosine metabolic enzymes and transporters) to maintain proper adenosine homeostasis. Notably, disruption of adenosine homeostasis in the brain under various pathophysiological conditions has been documented. In the past two decades, adenosine receptors (particularly A_1_ and A_2A_ adenosine receptors) have been actively investigated as important drug targets in major degenerative diseases. Unfortunately, except for an A_2A_ antagonist (istradefylline) administered as an adjuvant treatment with levodopa for Parkinson’s disease, no effective drug based on adenosine receptors has been developed for neurodegenerative diseases. In this review, we summarize the emerging findings on proteins involved in the control of adenosine homeostasis in the brain and discuss the challenges and future prospects for the development of new therapeutic treatments for neurodegenerative diseases and their associated disorders based on the understanding of adenosine homeostasis.

## Background

Adenosine is a purine nucleoside. It serves as a neurotransmitter and neuromodulator in the central nervous system (CNS). In addition, adenosine is an essential component of energy production and is utilized in all living cells. Adenosine can be produced during the catabolism of adenosine triphosphate (ATP), which is catalyzed by ectonucleotidases and endonucleotidases [145]. Extracellular and intracellular adenosine levels are modulated through equilibrative nucleoside transporters (ENTs) that bidirectionally transport adenosine across the plasma membrane down a concentration gradient and concentrative nucleoside transporters (CNTs) that transport adenosine into cells in a Na^+^-dependent manner against the concentration gradient [193]. Extracellular and intracellular adenosine play distinct roles in modulating various physiological functions (e.g., immune responses, blood–brain barrier (BBB) permeability, neuronal activity, and energy balance) in the CNS. Extracellular adenosine regulates intercellular signaling via adenosine receptors (A_1_R, A_2A_R, A_2B_R, and A_3_R) on the cell surface. In contrast, intracellular adenosine is important in the regulation of energy metabolism and DNA methylation [9, 35, 37, 48, 71, 198]. Disruption of adenosine homeostasis in the brain has been implicated in various pathophysiological dysfunctions, such as sleep disorders, cognitive impairment, and neuroinflammation [63, 198]. In the present review, we summarize the emerging role of dysregulated adenosine homeostasis in brain disorders with a specific focus on neurodegenerative diseases and possible new treatment developments based on these findings.

Most degenerative diseases are associated with abnormal aggregation of a disease-causing protein [203]. Specifically, the major pathogenic hallmarks of Alzheimer's disease (AD) are the accumulation of extracellular amyloid-β (Aβ) plaques and intracellular tau tangles [32]. Brains of patients with Parkinson's disease (PD) commonly contain Lewy bodies with misfolded α-synuclein [28]. Huntington's disease (HD) is caused by the formation of intracellular inclusions of mutant Huntingtin (mHTT), which contains an abnormal expansion of CAG repeats [169]. Amyotrophic lateral sclerosis (ALS) shows progressive loss of motor neurons with the accumulation of ubiquitinated TAR DNA-binding protein-43 (TDP-43) [166]. Although these degenerative diseases present with distinct clinical symptoms and affect different brain areas, poor energy homeostasis and mitochondrial dysfunction have been commonly documented [34, 57, 88, 129, 195, 233]. Furthermore, accumulating evidence also implicates dysregulated purine metabolism and abnormal expression of adenosine metabolism enzymes in these neurodegenerative diseases [8, 25, 90, 99, 114, 127, 217, 230]. Together, these findings suggest a possible association between purine metabolism and mitochondrial function in protein misfolding diseases. Specific details are discussed in the following sections.

### Adenosine metabolism enzymes

**Fig. 1:**
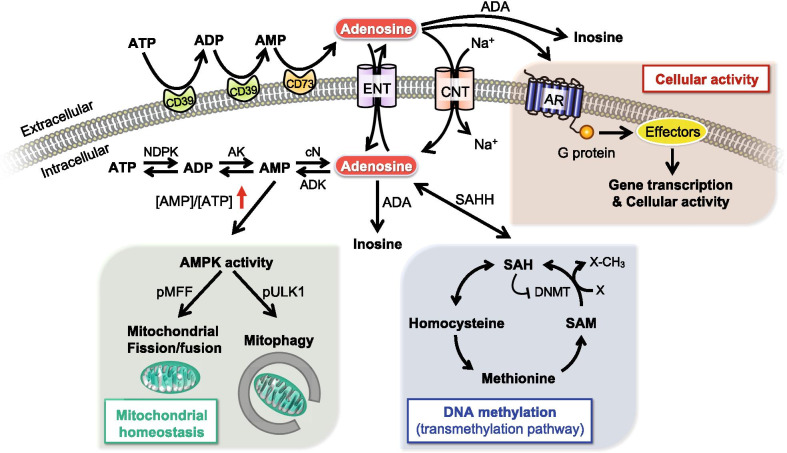
Regulatory components of the adenosinergic signaling pathway. Adenosinergic signaling is regulated by multiple molecules inside and outside of cells. In the extracellular compartment, ATP and ADP can be hydrolyzed into AMP by CD39. AMP is then converted into adenosine by CD73. Extracellular adenosine can either activate AR-mediated cellular signaling through G proteins and associated effectors, degrade into inosine by ADA, or be transported across the plasma membrane through Na^+^-independent passive nucleoside transporters (i.e., ENTs) or Na^+^-dependent active nucleoside transporters (i.e., CNTs). In the intracellular compartment, ATP can be hydrolyzed into ADP and AMP by NDPK and AK, respectively. Intracellular AMP can be further converted into adenosine by cN. Intracellular adenosine can either be degraded into inosine by ADA, transported to the extracellular compartment, or resynthesized into ATP by metabolic enzymes. During the adenosine metabolism process, the elevation of the AMP-to-ATP ratio can lead to the activation of AMPK, which further modulates mitochondrial homeostasis (e.g., mitochondrial fission/fusion or mitophagy) by changing the activity of associated enzymes (e.g., MFF or ULK1). Intracellular adenosine is also associated with DNA methylation by negatively regulating transmethylation pathways. In this pathway, SAM is demethylated to SAH by DNMTs, which subsequently enhances the level of DNA methylation. SAH is then hydrolyzed into adenosine and homocysteine by SAHH. Adenosine negatively regulates the activity of SAHH, which alters the SAH level and inhibits SAM-dependent DNMT activity. ADA, adenosine deaminase; ADK, adenosine kinase; AK, adenylate kinase; AMPK, AMP-activated protein kinase; ARs, adenosine receptors; CNTs, concentrative nucleoside transporters; ENTs, equilibrative nucleoside transporters; NDPK, nucleotide diphosphokinase; NT5C/cN, endonucleotidase; SAH, S-adenosyl-homocysteine; DNMTs, DNA methyltransferases; SAHH, S-adenosyl-homocysteine hydrolase; and SAM, S-adenosyl-methionine

The level of intracellular and extracellular adenosine is tightly regulated by multiple enzymes, including ectonucleotidases, endonucleotidases, adenosine deaminase (ADA), and adenosine kinase (ADK). In the extracellular compartment (e.g., synaptic cleft), two major ectonucleotidases (i.e., NTPDase1/CD39 and eN/CD73) are critical for the breakdown of ATP [261]. As shown in Fig. 1, ATP and adenosine diphosphate (ADP) can be hydrolyzed into adenosine monophosphate (AMP) by CD39. AMP is then converted into adenosine by CD73. Extracellular adenosine can be either degraded into inosine by ADA or transported across the plasma membrane through nucleoside transporters (i.e., ENTs and CNTs). In the intracellular compartment, adenosine metabolism is mediated by nucleotide diphosphokinase (NDPK), adenylate kinase (AK), endonucleotidase (e.g., cytosolic nucleotidase NT5C/cN), ADA, and ADK. Intracellular ATP can be hydrolyzed into ADP and AMP by NDPK and AK, respectively. Intracellular AMP can be further converted into adenosine by cN. Intracellular adenosine can either be degraded into inosine by ADA, transported to the extracellular compartment, or resynthesized into ATP by these metabolic enzymes (for recent reviews, see [27, 91]).

The roles of enzymes involved in adenosine metabolism in the CNS have been extensively investigated. CD39 and CD73 are known to control the balance between ATP and adenosine in the extracellular compartment. CD39/CD73-mediated ATP breakdown has been proposed to shift immune cells from a proinflammatory to an anti-inflammatory state [10]. This phenomenon was observed not only in peripheral immune cells but also in glial cells in the CNS. Recent studies have demonstrated that genetic removal of astrocytic CD73 blocks AMP-mediated cytokine release from astrocytes [258]. Badimon et al. [16] recently showed that microglial CD39/CD73-mediated ATP hydrolysis suppresses neuronal activity by activating A_1_ adenosine receptor (A_1_R) in a feedback regulatory manner. In addition, genetic removal of CD39 prevents the chronic social defeat stress (CSDS)-induced depression-like behavior of mice [60]. Similarly, ADA and ADK have also been implicated in neurodegenerative diseases. Suppression of ADA resulted in beneficial effects in a MPTP-induced Parkinsonism mouse model [114] but toxic effects on motor neurons in an ALS model established with a cocultured system of induced pluripotent stem cell (iPSC)-derived astrocytes and motor neurons [7]. Accumulating evidence suggests that ADK is an epigenetic modulator in both normal and disordered neurodevelopment [181, 206]. Together, these studies highlight the critical roles of adenosine metabolism in maintaining appropriate brain functions in health and diseases and suggest that adenosine metabolism enzymes may serve as potential drug targets for neurodegenerative diseases.

### Adenosine transporters

Nucleoside transporters act mainly on the transportation of adenosine across the plasma membrane and thus modulate adenosine homeostasis. To date, two major types of nucleoside transporters have been identified: (1) equilibrative nucleoside transporters (e.g., ENT1-4) and (2) concentrative nucleoside transporters (e.g., CNT1-3) [193]. ENTs are passive nucleoside transporters that are widely expressed in all tissues (including the brain). The main function of ENTs is facilitation of the bidirectional transportation of nucleosides (including adenosine) across membranes down a concentration gradient (Fig. 1). ENT1 and ENT2 are the most characterized adenosine transporters that are localized on the plasma membrane in the brain. Both ENT3 and ENT4 are pH-sensitive adenosine transporters that function in acidic environments. ENT3 is an intracellular enzyme on lysosomes that controls nucleoside homeostasis [112]. Similar to ENT1 and ENT2, ENT4 is located on the plasma membrane and modulates adenosine transportation across the membrane under acidotic conditions [18]. CNTs, on the other hand, are active nucleoside transporters with distinct expression profiles in various tissues. CNTs transport nucleosides into cells with an inward transmembrane Na^+^ gradient. The specific role of CNTs has been extensively discussed in recent reviews [91, 193] and therefore is not discussed here. In the current review, we focus on the current progress on understanding the roles of ENT1 and ENT2 in the CNS.

Previous studies have suggested that ENT1 and ENT2 play critical roles in the control of immune responses, mitochondrial activity, and neurological functions. Pharmacological inhibition or genetic deficiency of ENT1 or ENT2 showed beneficial roles in preventing lipopolysaccharide (LPS)-induced neuroinflammation [244], LPS-induced acute pulmonary inflammation [178], Pseudomonas aeruginosa–induced NLR family pyrin domain-containing 3 (NLRP3) inflammasome activation [45], and disease-associated neuroinflammation [46, 144]. In addition, genetic removal of ENT1 reduced essential astrocytic proteins (e.g., glial fibrillary acidic protein, excitatory amino acid transporter 2 (EAAT2), and aquaporin 4) critical for astrocyte functions [110]. In contrast, in endothelial cells, inhibition of ENT1/2 by dipyridamole prevented sustained adenosine exposure-induced mitochondrial dysfunction [135]. This finding is of great interest because Lai et al. had previously reported that human ENT1, but not mouse ENT1, is localized to mitochondria via a PE*X*N motif and functionally facilitates the transport of nucleoside drugs (e.g., fialuridine) into mitochondria [142, 147]. No further characterization of the role of ENT1 in mitochondrial functions has been recently reported; however, several mitochondrial proteins have been observed in the human ENT1 interactome network [21]. Further investigation is needed to dissect the potential functions and regulatory models of ENTs in different brain cells.

### Adenosine receptors

Adenosine signaling is executed by adenosine receptors on the plasma membrane. To date, four types of adenosine receptors (A_1_R, A_2A_R, A_2B_R, and A_3_R) have been identified and characterized. These receptors are G protein-coupled receptors (GPCRs) that modulate the adenylyl cyclase (AC)-cyclic adenosine monophosphate (cAMP)-protein kinase A (PKA) signaling pathway (Fig. 1). A_1_R and A_3_R are G_i/o_-coupled receptors that inhibit cAMP signaling, whereas A_2A_R and A_2B_R are G_s_-coupled receptors that stimulate cAMP signaling. A_1_R and A_2A_R are the major adenosine receptor subtypes in the brain and can be found in both neurons and glia. In many pathophysiological conditions tested, A_1_R and A_2A_R may have opposite effects in the regulation of cellular functions [145]. For example, synaptic transmission can be either enhanced by A_2A_R activation or inhibited by A_1_R activation. The functional roles of A_1_R and A_2A_R in many neurodegenerative diseases (including AD, HD, ALS, and PD) have been extensively investigated throughout the past two decades. The current progress has been summarized and discussed in detail in recent reviews [15, 91, 157] and thus is not elaborated here. Previously, A_2B_R and A_3_R were believed to exert only minor effects in the brain due to their low levels of expression and relatively low affinities for adenosine. Nonetheless, a few studies recently showed that A_2B_R participates in synaptic transmission and autoimmune neuroinflammatory reactions in the brain [56, 86, 94]. In addition, A_3_R may negatively modulate the functional properties of α-amino-3-hydroxy-5-methyl-4-isoxazolepropionic acid (AMPA) receptors in hippocampal neurons [70] and suppress microglial functions (including phagocytic activity and migration) [85]. Activation of A_3_R by MRS5980 improves traumatic brain injury-induced cognitive impairment [81]. These studies suggest that all four adenosine receptors are involved in the modulation of brain activity. Notably, genetic removal of all four adenosine receptors shortens the life span and causes the loss of adenosine-mediated hypothermia. Given that the overall physiology of these quadruple-knockout mice is similar to that of each of the four single-gene-knockout mice, the functions of these four adenosine receptors appear complementary but not synergistic [246]. To determine how the loss of all four adenosine receptors affects neuroplasticity and neuron-glia interactions, further investigation is required.

### Adenosine, AMP-activated protein kinase (AMPK), and energy homeostasis

Ample evidence suggests that adenosine homeostasis is important for cellular metabolism and has been implicated in the status of AMPK activation [46, 171, 192, 207]. AMPK is a key energy sensor and regulator in cells [108]. It is a heterotrimeric complex composed of three distinct subunits: α (catalytic; α1 and α2), β (scaffolding; β1 and β2), and γ (regulatory; γ1, γ2, and γ3) subunits [108]. The binding sites of adenine nucleotides on the γ subunit allow AMPK to sense the intracellular level of AMP, ADP, and ATP [245]. Under low-energy conditions, the AMP level is elevated, and the AMP/ATP ratio is thus increased, which facilitates the phosphorylation of AMPKα subunits at Thr^172^ and activates AMPK activity [222] (Fig. 1). In addition to sensing the cellular energy status, AMPK modulates multiple metabolic pathways to restore the energy status by inhibiting the anabolic pathway and stimulating the catabolic pathway [108]. AMPK serves as a key regulator of mitochondrial function by phosphorylating mitochondrial fission factor (MFF) and Unc-51-like autophagy-activating kinase 1 (ULK1), which affect mitochondrial dynamics (fission/fusion) and mitophagy, respectively [204, 219] (Fig. 1). This role of AMPK is interesting because mitochondrial dysfunction is a common feature of neurodegenerative diseases [34, 57, 129, 195].

Nonetheless, the role of AMPK in neurodegenerative diseases appears complex. Studies on different experimental models of various disease stages have reported seemingly contradictory conclusions. Assefa and colleagues reviewed recent progress in AD research and concluded that both positive and negative effects of AMPK activation have been found in AD [14]. Similarly, both activation and inhibition of AMPK have been found to be associated with beneficial effects on HD progression [107, 124, 125, 224]. In ALS, although suppression of AMPK has been demonstrated to produce beneficial effects in several experimental ALS models [153, 155, 156], studies have reported that AMPK activation may result in beneficial effects [58, 237]. As an explanation, most of the AMPK activators (e.g., metformin) tested were indirect activators and may have produced contradictory effects through AMPK-independent pathways. Moreover, different AMPK holoenzymes may regulate distinct types of machinery and therefore exert seemingly opposite functions in the brain. For example, recent studies have shown that AMPKα1 and AMPKα2 play detrimental and beneficial roles in memory functions, respectively [250, 262]. It is therefore vital to further evaluate the sensitivity of different AMPK subtypes to various AMPK activators and the exact roles of AMPK holoenzymes composed of different subunits in neurons and glia during the progression of neurodegenerative diseases.

### Adenosine, DNA methylation, and gene regulation

DNA methylation, a type of epigenetic modification, controls gene expression by preventing the binding of transcription factors to the promoter region of the corresponding gene [122, 238]. The dysregulation of DNA methylation has been implicated in neurodegenerative diseases [158]. For instance, the hypomethylation of APP promoter enhances Aβ level and AD pathology [242]. In addition, the hypomethylation of *TNF-α* promoter elevates its expression, which subsequently increases the vulnerability of dopaminergic neurons in PD [41, 196]. Interestingly, altered methylation of the 5’UTR region of *ADORA2A* reduces A_2A_R expression, which impairs adenosinergic signaling in the striatum of HD [226]. Methylation of the glutamate transporter (EAAT2) promoter contributes to the astroglial dysfunction in ALS [251]. These observations are of great interest because adenosine is also involved in the transmethylation pathway (Fig. 1), in which S-adenosylmethionine (SAM) is demethylated to S-adenosylhomocysteine (SAH) by DNA methyltransferases (DNMTs), subsequently enhancing the level of DNA methylation [50]. SAH is then hydrolyzed into adenosine and homocysteine by S-adenosylhomocysteine hydrolase (SAHH) [190]. Notably, this SAHH reaction is tightly and reversibly controlled by the concentrations of intracellular adenosine and homocysteine [190, 235, 241]. Specifically, adenosine negatively regulates the activity of SAHH, which further alters the SAH level. In addition, SAH can inhibit SAM-dependent DNMT activity, which forms a negative feedback regulation loop [120]. Thus, when the level of SAH is increased, DNA methylation is reduced [43]. Given that DNA methylation controls gene expression, adenosine homeostasis may contribute to the DNA methylation-mediated gene expression profile. Importantly, homocysteine has also been implicated in various diseases (including neurodegenerative diseases), which has been extensively reviewed elsewhere [104, 177, 210]. These studies collectively have shown that the cellular adenosine concentration can be regulated through the transmethylation pathway, while the adenosine concentration conversely may also influence the level of DNA methylation and gene expression profile. Proteins (such as ENTs and ADK) that control the cellular adenosine level are thus thought to play important roles in transmethylation pathway regulation and gene expression.

Previous genetic and pharmacological approaches have clearly demonstrated that adenosine modulates DNA methylation in a receptor-independent manner. For instance, genetic removal of ADK in the mouse forebrain leads to the elevation of intracellular adenosine and a lower level of DNA methylation. Specifically, chronic inhibition of ADK by an ADK inhibitor (5-iodotubercidin administered by intraperitoneal injection) results in the global reduction in DNA methylation in the hippocampus [241]. In addition, downregulation of ADK expression under hypoxic conditions leads to increased intracellular adenosine levels, enhanced DNA hypomethylation of the VEGFR2 promotor, and elevated proliferation of endothelial cells [247]. Interestingly, adenosine receptor genes can also be epigenetically modulated through adenosine-triggered DNA hypomethylation. Three CpG islands in the 5’ untranslated region (5’ UTR) of human *ADORA2A* have been identified and were shown to modulate the expression of A_2AR_ through DNA methylation [30, 31]. Together, these findings show crucial cross talk among factors influencing adenosine metabolism, the transmethylation pathway, and DNA methylation-dependent gene expression.

### Adenosine as a mediator of cross talk between neurons and glial cells

**Fig. 2:**
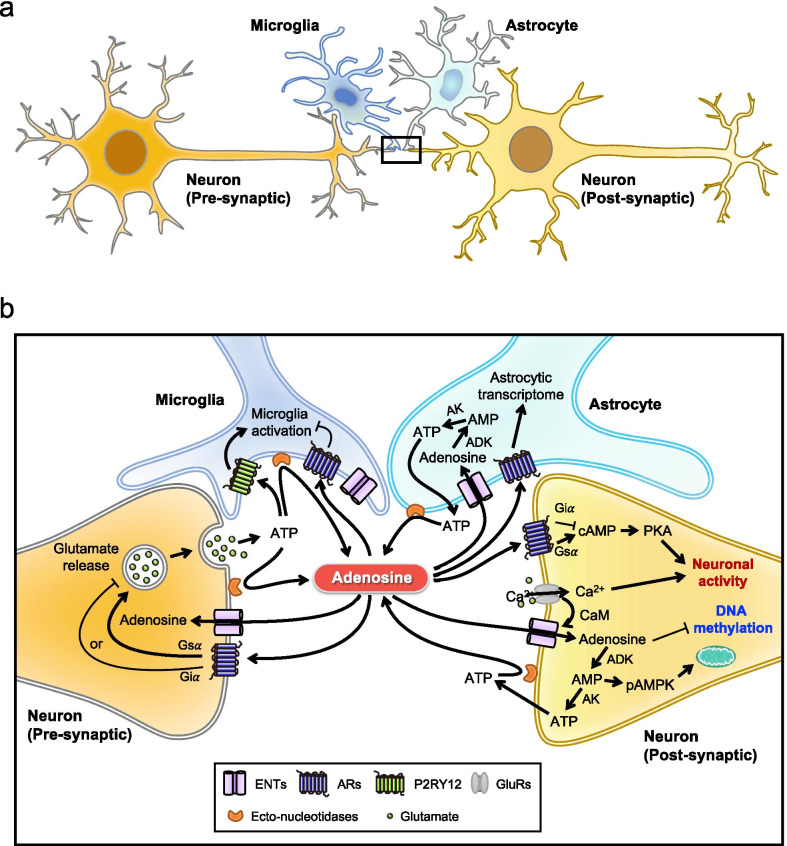
Regulatory roles of adenosine in neurons and glial cells. **a** Neuronal activity is modulated by the cross talk between neurons and glial cells (i.e., microglia and astrocytes). **b** Adenosine is an important modulator in the brain. In neurons, upon activation, glutamate and ATP are released from the presynaptic terminal to activate receptors in the synapse, which modulates neuronal activity, calmodulin-mediated regulation of ENT affinity to transport adenosine, and adenosinergic pathways (as shown in Fig. 1). In microglia, released ATP can attract microglia by activating P2RY12. ATP can be quickly hydrolyzed into AMP and adenosine by ectonucleotidases (e.g., CD39 and CD73), which are enriched on the membrane of microglia and expressed on neurons and astrocytes. The resultant adenosine then activates neuronal ARs (e.g., A_1_R and A_2A_R) to suppress or facilitate the further release of neurotransmitters from the presynaptic terminal. In astrocytes, adenosine can also regulate the metabolism of astrocytes and the astrocytic transcriptome via the activation of AR (i.e., A_2B_R). ADK, adenosine kinase; AK, adenylate kinase; AMPK, AMP-activated protein kinase; ARs, adenosine receptors; CaM, calmodulin; ENTs, equilibrative nucleoside transporters; GluRs, glutamate receptors; and P2RY12, purinergic receptor P2Y, G-protein coupled, 12

Glial malfunction, which causes abnormal interaction between neurons and glia, has been shown to contribute significantly to neurodegenerative diseases [213]. Adenosine is known to mediate the cross talk between neurons and glia (Fig. 2). Upon neuronal activation, neurotransmitters (e.g., glutamate and ATP) are released from the presynaptic terminal to activate receptors in the synapse [191]. The released ATP can attract microglia through the activation of purinergic receptor P2Y, G-protein coupled,12 (P2RY12), which also mediates the establishment of somatic microglia-neuron junctions [59, 106]. Because ectonucleotidases (e.g., CD39 and CD73) are enriched on the membrane of microglia, the ATP released from neurons can be quickly hydrolyzed into AMP and adenosine. The resultant adenosine then activates neuronal A_1_R to suppress the further release of neurotransmitters [16]. Neuronal activity can also regulate the metabolism of astrocytes. Astrocytic genes associated with glucose metabolism and lactate export are upregulated via the activation of the A_2B_R-cAMP-PKA-cAMP-response element-binding protein (CREB) axis, which controls the interaction between neurons and astrocytes [6, 105]. As described in the previous section, adenosine that is abundant in the extracellular compartment can be transported into both astrocytes and neurons via ENTs or hydrolyzed into inosine by ADA to maintain adenosine homeostasis. As glutamate excitotoxicity and neuroinflammation are common features of neurodegenerative diseases [118, 150, 234], these neuron-microglia interactions are critical because excessive activation of neurons can disrupt neuronal functions, shorten their survival and cause severe inflammation by significantly elevating ATP release and altering adenosinergic signaling [72, 184]. In AD, the level of P2RY12 in microglia near amyloid plaques and tau tangle-containing plaques is low [164, 231], suggesting a loss of negative feedback regulation between microglia and neurons. In addition, previous studies have revealed cell-specific expression and dysregulation of genes involved in purinergic signaling and metabolism, which may greatly alter the cross talk between neurons and glia in degenerative diseases [8, 145, 197, 208, 211].

### Adenosine homeostasis in neurodegenerative diseases

#### Alzheimer’s disease (AD)

AD is characterized by progressive memory loss and cognitive decline and has become the major neurodegenerative disease in aging populations of modern societies. The two key pathogenic hallmarks of AD are the accumulation of extracellular plaques composed of β-amyloid (Aβ) and intracellular neurofibrillary tangles containing hyperphosphorylated tau proteins [32]. Both pathologies are associated with synaptic loss and neuroinflammation, which cause cerebral dystrophy and cognitive impairments during AD progression [49, 146, 209].

Abnormal adenosine homeostasis has been reported earlier in the brains of AD patients [8, 44]. Altered expression of adenosine receptors (including A_1_R and A_2A_R) has been found in the frontal cortex of AD patients and AD mice (APP/PS1) [5, 144, 225]. The dysregulated adenosinergic system has long been considered a possible target for the development of new treatments for AD [46, 143, 144, 174, 186, 187]. In particular, suppression of A_2A_R or A_1_R provides beneficial effects on different molecular aspects related to the cognitive functions of AD mice [67, 79, 143, 187]. Nonetheless, the beneficial effects of small molecules targeting adenosine receptors seem moderate and may be effective only in specific treatment windows [187]. Because recent progress on this topic has been extensively reviewed [15, 74], no further elaboration on the function and regulation of adenosine receptors in AD is presented here.

Here, we focus on several proteins involved in adenosine metabolism that may serve as novel drug targets for AD; namely, ENT1, which is the most well-characterized adenosine transporter in the brain. Two recent studies reported that chronic treatment with an orally active, BBB-permeable small ENT1 inhibitor (J4) attenuated impaired cognitive function, inferior neuroplasticity, and elevated neuroinflammation in two AD mouse models (APP/PS1 and THY-Tau22) [46, 144]. Other enzymes involved in adenosine metabolism (ADA, ADK, CD39 and CD73) are also abnormally affected in AD, similar to other neurodegenerative diseases (Table 1). In the brains of patients with early stage AD, ADA activity is relatively low in the frontal cortex and high in the temporal cortex, suggesting brain area-specific regulation of adenosine homeostasis in AD [8]. In the hippocampus of an experimental model of tauopathy (THY-Tau22), the levels of ADA, CD39 and CD73 were all higher than those of WT mice (Table 1). Partial blockade of ENT1 by J4 effectively normalized these levels and thus stabilized the aberrant adenosine metabolism in tauopathy [46]. Another interesting finding showed that astrocytes surrounding amyloid deposits and tangle-containing neurons contain abundant ADK [25], suggesting that ADK inhibitors may have therapeutic effects. On the other hand, the elevated CD73 level in AD is known to contribute to the impairment of synaptic plasticity and low cognitive function induced by β-amyloid plaques. Since the detrimental effect of β-amyloid plaques is greatly reduced in the absence of this ecto-5′-nucleotidase, CD73 is also a novel drug target for AD [93].

**Table 1:** Regulation of adenosine homeostasis-related proteins in neurodegenerative diseases

Disease	Component	Effect	Model	Clinical (C)/Preclinical (P)	References
AD	ADA	Up	Activity in AD patients	C	[8]
		Up	Thy-Tau22 mouse model	P	[46]
	ADK	Up	Protein level in AD patients	C	[25]
	CD39	Up	APP/PS1 mouse model	P	[19]
		Up	Thy-Tau22 mouse model	P	[46]
	CD73	Up	Activity in β-amyloid (Aβ_1-42_)-based mouse model of early AD	P	[93]
		Up	Thy-Tau22 mouse model	P	[46]
	AMPK	Up	AMPKα1 in AD patients	C	[262]
		Down	AMPKα2 in AD patients	C	[262]
		Up	AMPKα1 in Tg19959 mouse model	P	[262]
		Up	Activity in AD patients	C	[162]
		Up	Activity in APP/PS1 mouse model	P	[162]
		Up	Activity in Thy-Tau22 mouse model	P	[46]
		Down	Activity in APP/PS1 mouse model	P	[232]
		Down	Activity in STZ-treated mouse model	P	[232]
PD	ADA	Up	Transcript level in the frontal cortex of PD patients at stages 3–4	C	[90]
		Up	Activity in the serum from PD patients	C	[51, 52]
		Up	Activity in the striatum of MPTP mouse model	P	[114]
	CD73	Up	Transcript level in the frontal cortex of PD patients at stages 5–6	C	[90]
		Up	Protein level in striatal synaptosome of a 6-OHDA rat model	P	[38]
		Up	Protein level in 6-OHDA-treated differentiated SH-SY5Y cells	P	[38]
	AMPK	Down	Total and phosphorylated levels in the substantia nigra of MPTP-treated mice and in MPP^+^-treated PC12 cells	P	[215]
		Unchanged	Total and phosphorylated levels in the substantia nigra of MPTP-treated mice	P	[249]
		Up	Phosphorylated level in the substantia nigra of MPTP-treated mice	P	[149]
		Up	Phosphorylated level in the substantia nigra of patients with dementia with Lewy bodies and of MPTP-treated and α-synuclein-overexpressing mice	P	[126]
HD	ENT1	Up	HD patients	C	[99]
		Up	R6/2 mouse model	P	[127]
		Up	Hdh150Q mouse model	P	[127]
		Up	zQ175 mouse model	P	[99]
	ENT2	Up	R6/2 mouse model	P	[127]
		Up	Hdh150Q mouse model	P	[127]
	ADA	Up	Activity in HEK 293 T cell line transfected with mutant exon 1 of the HTT gene	P	[217]
	ADK	Up	R6/2 mouse model	P	[127]
	CD39	Down	Activity in HEK 293 T cell line transfected with mutant exon 1 of the HTT gene	P	[217]
	CD73	Down	Activity in HEK 293 T cell line transfected with mutant exon 1 of the HTT gene	P	[217]
	AMPK	Up	Activity in HD patients	C	[125]
		Up	Activity in R6/2 mouse model	P	[125]
ALS	ADA	Down	ALS patients-derived astrocyte	P	[7]
	ADK	Up	Activity in reactive astrocytes of ALS patients	C	[25]
	CD39	Down	Transcript level in ALS patients	C	[33]
		Down	Transcript and protein level in SOD1(G93A) mouse model	P	[33, 230]
	CD73	Up	SOD1(G93A) mouse model	P	[87]
	AMPK	Down	ALS patients-derived BM-MSC	P	[256]
		Up	Activity in ALS patients	C	[155, 156]
		Up	Activity in SOD1(G93A) mouse model	P	[153, 257]
		Up	Activity in TDP-43 mouse model	P	[155]
Epilepsy	ENT1	Up	Protein level in neocortex of patients with temporal lobe epilepsy	C	[248]
		Up	Protein level in the hippocampus of a pilocarpine-induced rat model	P	[248]
	ADK	Up	Protein level in temporal lobe of patients with epilepsy	C	[13, 165]
		Up	Protein level in the hippocampus and astrocytes of a kainite mouse model	P	[96]
		Up	Protein level in the hippocampus, cortex, and astrocytes of rats with status epilepticus	P	[13]
	AMPK	Down	Protein level in the neocortex of epileptic patients	C	[252]
		Down	Protein level in the hippocampus of pentylenetetrazol- and kainic acid-induced mouse models	P	

6-OHDA, 6-hydroxydopamine; ADA, adenosine deaminase; ADK, adenosine kinase; AMPK, AMP-activated protein kinase; BM-MSC, Bone marrow mesenchymal stem cells; ENT1, equilibrative nucleoside transporter 1; MPTP, 1-methyl-4-phenyl-1,2,3,6-tetrahydropyridine; MPP^+^, 1-methyl-4-phenylpyridinium; STZ, Streptozotocin

The abnormal adenosine metabolism observed in the brain with AD implies that neuronal energy status may be similarly dysregulated. Moreover, mitochondrial impairment is a pathological feature of AD, which is thought to further impair energy homeostasis in the brain. AMPK is a molecular sensor of cellular energy status, and abnormal AMPK activation has been reported in AD brains [46, 80, 162, 228]. An early study suggested that the suppression of overactivated AMPK using a selective AMPK inhibitor (compound C) leads to the improvement of AD-associated symptoms [162]. Targeting abnormally regulated AMPK signaling as a therapeutic strategy for AD has therefore attracted considerable attention. Notably, both AMPK subtypes (α1 and α2) can be found in the brain, and they not only perform very different functions but also undergo distinct regulation in AD. Compared with that in the brains of normal subjects, the level of AMPKα1 was significantly increased in the hippocampi of AD patients and Tg19959 mice, while the level of AMPKα2 was reduced. Genetic ablation of AMPKα1 produces beneficial effects on cognitive functions in two AD mouse models (Tg19959 and APP/PS1, [262]) (Table 2). In contrast, selective removal of AMPKα2 in the hippocampus reduces dendritic spine density and neuronal plasticity, probably because of the disturbance in protein translation [250]. The subtype-specific function of AMPKs may contribute to the seemingly controversial findings. For example, treatment with metformin (a known AMPK activator) ameliorates synaptic deficiency and improves the memory function of AD mice (APP/PS1) [236]. However, many AMPK activators that have been tested in experimental models of AD also have AMPK-independent functions. Further investigations are necessary to delineate the detailed regulation and function of AMPK subtypes in AD brains to make therapeutic intervention possible.

**Table 2:** Adenosine homeostasis-related proteins as drug targets in neurodegenerative diseases

Disease	Target	Effect	Model	References
AD	ENT1	Enhanced spatial memory Enhanced synaptic transmission	Pharmacological inhibitor (J4) of ENT1 in APP/PS1 mouse model	[144]
		Enhanced spatial memory Reduced AMPK activity Reduced ADA, CD39 and CD73 levels	Pharmacological inhibitor (J4) of ENT1 in Thy-Tau22 mouse model	[46]
	CD73	Enhanced memory and synaptic function	Genetic knockout of CD73 in a β-amyloid (Aβ_1-42_)-based mouse model of early AD	[93]
	AMPK	Enhanced long-term potentiation (LTP)	Pharmacological inhibitor (compound C) of AMPK in APP/PS1 mouse model	[162]
		Enhanced spatial memory Reduced tau acetylation level	AAV-mediated AMPK overexpression in APP/PS1 mouse model	[232]
		Enhanced spatial memory	AAV-mediated AMPK overexpression in STZ-treated mouse model	[232]
		Enhanced spatial memory	Pharmacological activator of AMPK (metformin) in APP/PS1 mouse model	[236]
		Enhanced learning and memory Enhanced cognitive function	Genetic repression of AMPKα1 in Tg19959 mouse model	[262]
		Enhanced learning and memory Enhanced cognitive function	Genetic repression of AMPKα1 in APP/PS1 mouse model	[262]
PD	ADA	ADA inhibition alleviated neuroinflammation, dopamine deficit, dopaminergic neuronal loss, and motor impairment	MPTP-induced mouse model	[114]
		ADA inhibition strengthened the protective effects of A_2A_R antagonist	MPTP-induced mouse model	[114]
		ADA application prevented 6-OHDA-induced neurotoxicity	6-OHDA-treated differentiated SH-SY5Y cells	[38]
	CD73	Microglial-mediated inflammation, dopaminergic neuronal loss, and motor deficits were partially attenuated in CD73 KO mice	MPTP-induced mouse model	[175]
		CD73 blockade prevented 6-OHDA-induced neurotoxicity	6-OHDA-treated differentiated SH-SY5Y cells	[38]
		Central administration of a CD73 inhibitor attenuated dopamine loss, dopaminergic neuronal death, motor dysfunction, and memory decline	6-OHDA-induced rat model	[38]
	AMPK	AMPK activation protected against α-synuclein accumulation, mitochondrial dysfunction, production of ROS, and neuronal death	α-Synuclein-, 6-OHDA-, and MPTP-induced models	[64, 103]
HD	ENT1	Enhanced survival	Genetic knockout of ENT1 in R6/2 mouse model	[127]
		Enhanced survival Improved motor function	Pharmacological inhibitor (JMF1907) of ENT1 in R6/2 mouse model	[127]
	AMPK	Enhanced survival	Pharmacological activator of AMPK (metformin) in R6/2 mouse model	[163]
		Reversed early cortical network dysfunction	Pharmacological activator of AMPK (metformin) in Hdh150Q mouse model	[12]
		Improved motor and neuropsychiatric function	Pharmacological activator of AMPK (metformin) in zQ175 mouse model	[205]
		Improved cognitive function	Pharmacological activator of AMPK (metformin) in HD patients	[107]
ALS	ENT1	Reduced AMPK activity Improved motor function	Pharmacological inhibitor (JMF1907) of ENT1 in TDP-43 mouse model	[155]
	AMPK	Increased differentiation rate	Pharmacological activator of AMPK (Resveratrol) in ALS patients-derived BM-MSCs	[256]
		Delayed disease progression	Pharmacological activator of AMPK (Latrepirdine) in SOD1 (G93A) mouse model	[58]
		Improved behavioral phenotypes Enhanced motor neuron survival	Pharmacological activator of AMPK (metformin) in C9orf72 ALS/FTD BAC mouse model	[263]
		Reduced AMPK activity Enhanced motor neuron survival Delayed disease progression Extended the lifespan	A high-fat diet in SOD1 (G93A) mouse model	[257]
Epilepsy	ENT1	Intrahippocampal injection of an ENT1 inhibitor ameliorated seizure severity and prolonged the onset latency	Pilocarpine-induced rat model	[248]
		Intraperitoneal injection of ENT1 inhibitors exhibited anti-seizure activities	Maximal electroshock-, pentylenetetrazol-, or kindling-induced mouse models	[111]
	ADK	An ADK inhibitor suppresses seizure activity	Kainic acid-induced mouse model	[96]
		Genetic reduction of ADK reduced seizure activity and neuronal injury	Kainic acid-induced mouse model	[152]
	AMPK	AMPK activation by metformin decreased mTOR phosphorylation and seizure activity	Pilocarpine-induced rat model	[172]
		AMPK activation by metformin reduced the mortality and the duration of seizures	Pentylenetetrazol and kainic acid-induced mouse models	[252]
Sleep disturbances	ENT1	Decreased sleep duration Decreased extracellular adenosine	Genetic knockout of ENT1 mouse model	[132]
	ADK	Decreased sleep duration	Transgenic overexpression of ADK mouse model	[189]
	CD73	Decreased sleep duration	Genetic knockout of CD73 mouse model	[260]
	AMPK	Decreased sleep depth	Pharmacological inhibitor (compound C) of AMPK in wild-type mouse model	[53]
		Enhanced sleep depth	Pharmacological activator (AICAR) of AMPK in wild-type mouse model	[53]

6-OHDA, 6-hydroxydopamine; ADA, adenosine deaminase; ADK, adenosine kinase; AICAR, 5-aminoimidazole-4-carboxamide ribonucleotide; AMPK, AMP-activated protein kinase; M-MSC, Bone marrow mesenchymal stem cells; ENT1, equilibrative nucleoside transporter 1; KO, knockout; MPTP, 1-methyl-4-phenyl-1,2,3,6-tetrahydropyridine; ROS, reactive oxygen species; STZ, Streptozotocin

#### Parkinson's disease

Parkinson’s disease (PD) is a common chronic neurodegenerative disease that affects approximately 1% of the elderly population. It is characterized by progressive loss of dopaminergic neurons in the substantia nigra, which directly leads to dopamine deficiency in the striatum and subsequent impairment of motor function. The current therapies rationally aim to normalize dopaminergic levels through specific treatments, such as the administration of a dopamine precursor (levodopa or L-dopa) and dopamine receptor agonists. Catechol-O-methyltransferase inhibitors, which prevent peripheral breakdown of levodopa, are clinically beneficial as adjuvants to levodopa therapy. In addition, inhibitors of monoamine oxidase type B, which suppress the degradation of endogenous dopamine, are also beneficial as a monotherapy or an adjuvant treatment with levodopa for early stage PD [140]. However, although many therapies for PD symptoms are available, these drugs are effective only at the early stage of the disease and are unable to slow or cure the disease. Moreover, long-term dopamine replacement therapy worsens unwanted side effects, particularly involuntary abnormal movements, such as those associated with dyskinesias and dystonia. New therapeutic targets for PD are therefore urgently needed.

The interaction between adenosine and dopamine signaling pathways has been extensively investigated and reviewed previously [183, 218]. Much attention has been focused on the roles of A_2A_R in the modulation of dopaminergic signaling because this adenosine receptor is highly enriched in the basal ganglia (including the striatum) and forms heteromeric complexes with dopamine receptor 2 (D_2_R). Nonselective adenosine receptor antagonists, caffeine and theophylline, were found to alleviate classical symptoms in animal models of PD. In addition to D_2_R, A_2A_R also interacts with A_1_R, metabotropic glutamate receptor-5, cannabinoid receptor type-1, and serotonin receptor 1A, modulating striatal neuron activity and motor performance [11, 24, 218]. Accordingly, it has been shown that specific A_2A_R antagonists attenuate motor impairment in a wide variety of PD models by reducing dopamine depletion, loss of dopaminergic neurons, production of reactive oxygen species, and aggregation of α-synuclein [218]. Among these A_2A_R antagonists, istradefylline (KW6002) has been approved as an adjunct treatment with levodopa/carbidopa in PD patients. More importantly, the treatment may not exacerbate the dyskinetic response [98]. The outcomes and safety profiles of many other A_2A_R antagonists have not met expectations [182, 218].

The success of istradefylline encourages the development of novel approaches targeting adenosine homeostasis as a nondopaminergic strategy for PD treatment. Aberrant gene expression involved in purine metabolism, such as ADA and CD73 expression, has been reported in PD-affected brain regions [90, 221] (Table 1), suggesting a primary manifestation or compensatory effects of altered purine metabolism in PD. Consistently, metabolites in purine metabolism pathways were found to be altered in the MPTP-based mouse model of PD [114]. These observations support the idea that the impairment of adenosine homeostasis is a rational target for PD pathogenesis. A hypothesis suggesting that inhibition of ADA with deoxycoformycin significantly ameliorates motor disability, dopamine depletion, and the death of dopaminergic neurons in PD mice has been supported. In addition, cotreatment with an ADA inhibitor and istradefylline showed additive antiparkinsonian activities [114]. Moreover, this combination treatment reduced microglial activation and overproduction of proinflammatory cytokine tumor necrosis factor-α in the substantia nigra and striatum, respectively [114] (Table 2). In contrast, removing extracellular adenosine with ADA was shown to prevent 6-hydroxydopamine (6-OHDA)-induced toxicity in a dopamine-differentiated SH-SY5Y cell model [38] (Table 2). The roles of ADA in the pathogenesis and intervention of PD remain to be clarified.

Extracellular adenosine can be produced through the CD73-mediated pathway. Genetic deletion of CD73 reduced the level of adenosine in the striatum but not in the cortex or hippocampus, showing a region-specific role of CD73 in adenosine formation [175]. With regard to PD, genetic deletion of CD73 modulated microglial-mediated responses, including reducing the release of proinflammatory cytokines in LPS-challenged microglia and increasing microglial process extension and mobility in an acute MPTP mouse model [175]. The role of CD73 in PD pathogenesis was also evaluated in a subacute MPTP model, with the results showing that, in addition to the modulation of neuroinflammatory responses, CD73 knockout partially prevented MPTP-induced dopaminergic neuron loss and motor impairment [175] (Table 2). The underlying mechanism has been shown to be associated with A_2A_R overactivation and unbalanced dopamine signaling in the nigrostriatal pathway [38, 175]. These findings support not only the supposition that A_2A_R activation plays a detrimental role in PD but also the idea that the development of CD73 inhibitors and the combined therapy of CD73 inhibitors with A_2A_R antagonists are worthy of further investigation.

AMPK activation has been shown to be neuroprotective in several disease models, including PD models. The downstream signaling pathways of AMPK have been implicated in the regulation of energy homeostasis, mitochondrial functions, macroautophagy, oxidative stress, and inflammatory responses. All of these pathways are relevant to the pathophysiology of PD. Accordingly, AMPK activation has been demonstrated to alleviate two major pathological hallmarks of PD, the degeneration of nigrostriatal dopaminergic neurons and the accumulation of α-synuclein in Lewy bodies in different PD model systems, including cell culture, fly, and rodent models (Table 2). Several excellent review articles have been devoted to these findings [64, 89, 103]. Among the AMPK activators, resveratrol and metformin have been tested the most extensively in PD models. However, the detrimental effects of activating AMPK have also been reported [64]. For example, AMPK overactivation may contribute to neuronal atrophy and progressive degeneration of dopaminergic neurons in a 6-OHDA-induced PD model [133]. Therefore, for the treatment of PD, the extent and duration of AMPK modulation should be carefully evaluated. On the other hand, identifying a more specific target in AMPK-related signaling pathways may be more favorable.

#### Huntington’s disease (HD)

HD is a dominant genetic neurodegenerative disease caused by an expansion of the CAG repeat in exon 1 of the *Huntingtin* gene. This expanded CAG repeat encodes a polyglutamine (polyQ) stretch in the Huntingtin protein (mHTT) and causes the accumulation of mHTT in neurons, which causes neuronal toxicity and death. The clinical presentations of HD include the progression of chorea, motor dysfunction, psychiatric abnormalities, and cognitive impairment, with the striatum and cerebral cortex the most affected brain areas [169]. Because HD is a monogenic disease, in the past two decades, excellent genetic mouse models have been established to support various mechanistic studies of HD pathogenesis. Nonetheless, at this time, drugs are available only for symptom relief, not for disease modification [39, 154].

The functions of two adenosine receptors (A_1_R and A_2A_R) have been evaluated as drug targets in HD [23, 47, 145]. The expression of A_1_R is reduced, while its ability to suppress presynaptic glutaminergic transmission is enhanced in HD mice. In addition, treatment with an A_1_R agonist (N6-cyclopentyladenosine) reversed mHTT-evoked neuronal toxicity. Compared with that of healthy subjects, the expression level of A_1_R was also found to be lower in HD patients in the symptomatic stage [167], suggesting that A_1_R may be a drug target [84]. A_2A_R is highly enriched in the striatum, the brain area most affected by HD, and therefore has attracted considerable attention in the past decade. Most likely because of mHTT-mediated toxicity induced during HD progression, a gradual loss of A_2A_R has been reported in patients and in mouse models of HD [99, 145]. Chronic treatment of HD mice (R6/2) with several agonists (CGS21680, T1-11) of A_2A_R markedly delayed disease progression and reduced the accumulation of the disease-causing protein (mHTT) [47, 54, 113]. Genetic removal of A_2A_R in HD mice (N171-82Q) also greatly worsened the motor performance and survival of the HD mice [176]. During disease progression, the expression of A_2A_R is known to gradually decrease in striatal medium spiny neurons (MSNs) and may contribute to the degeneration of MSNs in HD. The loss of A_2A_R in the late disease stage suggests that the therapeutic window for A_2A_R agonists might exist in the early symptomatic stage [145].

Abnormal adenosine metabolism has also been observed in several mouse models (R6/2, zQ175) and humans with HD. In the striatum, the expression levels of two ENTs are elevated, while brain adenosine tone is diminished (Table 1) [99, 127]. Suppression of ENT1 by genetic or pharmacological approaches both improved motor functions and prolonged the survival of HD mice (Table 2) [127]. Interestingly, the expression of mHTT enhances the activity of ADA while suppresses those of CD39 and CD73 in a cell model (HEK293) [217]. Moreover, the amount of ADK transcript was found to be upregulated in HD mice (R6/2) [127] (Table 1). These findings collectively indicate that adenosine homeostasis is dysregulated at multiple levels and that proteins controlling adenosine homeostasis (e.g., ENT1) may serve as good drug candidates to delay HD progression.

As mentioned above, adenosine homeostasis is closely associated with cellular energy status. Moreover, the expression of mHTT has long been known to damage mitochondria [212], which may further impair the energy supply to neurons in HD. Consistently, the expression of mHTT in a cell model causes a reduction in ATP [217]. In the brains of HD mice (R6/2) in the late disease stage and HD patients, abnormal overactivation and translocation of AMPK-α1 in MSNs exert detrimental effects [124, 125] (Table 1). Activation of AMPK-α1, therefore, is likely to worsen neuronal survival in HD. Nonetheless, treatment of HD mice (Hdh^150Q^ knock-in, R6/2, and zQ175) with metformin, a first-line antidiabetic drug that can activate both AMPK-dependent and AMPK-independent pathways, produced beneficial effects [12, 107, 163, 205]. As discussed above, it is important to evaluate whether the beneficial effect of metformin is AMPK-dependent. In addition, the contribution of AMPK isoforms to the effect of metformin (or other AMPK activators) and HD pathogenesis at different disease stages requires further investigation.

#### Amyotrophic lateral sclerosis (ALS)

ALS is a rare motor neuron disease caused by the degeneration of upper and lower motor neurons, resulting in progressive paralysis, muscle wasting, and early death [240]. Most ALS cases are sporadic (sALS, ~ 90%), and approximately 10% of ALS patients (fALS) have mutations in a group of more than 50 genes associated with ALS (including *C9orf72*, *SOD1*, *TARDBP*, *FUS,* and *HuR*) [130, 173]. Most likely due to the complex etiology of ALS, only a handful of drugs (e.g., edaradone, riluzole, and dextromethorphan) with limited therapeutic effects are currently available. Although promising approaches targeting various machineries (e.g., neuroinflammation, neuronal hyperexcitability, energy homeostasis, and mitochondrial dysfunction) have been actively pursued, no treatment that can halt or reverse the progression of ALS is currently available [131].

Abnormal adenosine homeostasis has long been observed in ALS. Specifically, the level of adenosine in the cerebrospinal fluid of ALS patients is higher than that of normal subjects [255]. Consistently, the expression levels of ADA in the neurons and astrocytes differentiated from the iPSCs of ALS patients were much lower than those obtained from normal subjects. Previous studies have also revealed the upregulation of ADK activity and the suppression of CD39 in the spinal cord of ALS patients and a mouse model of ALS (SOD1(G93A)) [25, 33, 230]. Collectively, these studies document abnormalities in adenosine homeostasis, which may weaken the ability of motor neurons in the spinal cord of ALS patients to respond to energy stress.

Not only is the adenosine level in the cerebrospinal fluid of ALS patients higher, but the expression of A_2A_R in the spinal cord of ALS patients also appears to be elevated [185, 227]. In a mouse model of ALS (SOD1(G93A)), A_2A_R appeared to be dynamically regulated in the spinal cord [185, 200]. Nonetheless, treatments targeting adenosine receptors produce seemingly inconsistent effects. For example, treatment of SOD1(G93A) mice with a nonselective adenosine receptor antagonist (caffeine) worsened their survival without affecting bodyweight or motor function [200]. Surprisingly, although the administration of an A_2A_R-selective antagonist (KW6002) and partial genetic ablation of A_2A_R both delayed diminished grip strength and attenuated the shortened lifespan of SOD1(G93A) mice, complete removal of A_2A_R showed no effect [185]. In contrast, treatment of SOD1(G93A) mice with a specific A_2A_R agonist (CGS2168) greatly delayed the onset of impaired rotarod performance and protected against the loss of motor neurons without affecting overall survival [254]. The treatment window of A_2A_R activation may have been narrow in this particular ALS mouse model. Interestingly, in another ALS mouse model (Tg-TDP-43), chronic treatment with another A_2A_R agonist (JMF1907) significantly improved motor functions by suppressing the overactivation of AMPK-α1 in the spinal cord [155]. In the future, the role of A_2A_R in ALS certainly needs further investigation, particularly with respect to disease stage, the choice of preclinical model, and route of administration.

Another emerging interest in ALS is the role of AMPK. Several studies have reported that AMPK is activated in motor neurons in the spinal cord of human ALS patients and several experimental models of ALS and that it plays a detrimental role in the survival of motor neurons [153, 155]. Importantly, the abnormal activation of AMPKα1 mediates the mislocalization of TDP-43 from the nuclei to the cytoplasmic region, an early event in ALS pathogenesis (Table 1) [155]. This overactivation of AMPK and mislocalization of several RNA-binding proteins (including TDP-43 and HuR) can be suppressed by inducing A_2A_R activation with one of several BBB-permeable agonists (JMF1907 and T1-11) via the cAMP/PKA-dependent pathway [141, 155, 156]. In addition, activation of AMPK in the spinal cord of SOD1-(G93A) mice was markedly enhanced by diet restriction, worsening symptom onset (as assessed by grip strength), motor neuron loss, and survival. A high-fat diet not only reduced the overactivation of AMPK but also produced marked beneficial effects, probably through an HSP90-dependent pathway [257]. These findings obtained in preclinical studies are consistent with those of a recent clinical study showing that a high-calorie fatty diet produced a marked survival benefit in ALS patients with fast progression [160]. Collectively, the data show that suppression of AMPK by various means may be a possible therapeutic strategy for ALS. Notably, treatments of ALS mice with several known AMPK activators (e.g., resveratrol, latrepirdine, and metformin) were shown to delay disease progression and enhance the survival of motor neurons [58, 256, 263]. As discussed above, most agents tested in preclinical studies activated not only AMPK but also other AMPK-independent pathway(s). It will be of great interest to determine whether the beneficial effects of these agents are mediated solely by AMPK and to characterize the sensitivity of AMPK subtypes to these agents.

#### Neurodegenerative disease-associated epilepsy

Epilepsy is a common brain disorder, which can be triggered by multiple pathological factors. Despite being a neuroelectrical disorder, epilepsy shares several pathological features (such as neurodegeneration, astrogliosis, and BBB abnormalities) with multiple neurodegenerative diseases [82], and is frequently observed in patients with neurodegenerative diseases [82, 194]. These observations suggest that neurodegenerative pathways can be the potential stimuli of epileptic seizures. In addition, early recognition and closer management are needed to minimize the morbidity and mortality resulting from seizures in patients with neurodegenerative diseases. Numerous drugs have been approved for the management of epilepsy based on the strategy of decreasing the electrical activity of the neuronal connection. The mechanisms of anti-epileptic drugs include the blockade of sodium channels/calcium channels/glutamate receptors, enhancement of γ-aminobutyric acid (GABA) receptor activity, and inhibition of GABA degradation/reuptake. However, approximately 30% of patients with seizures are respond poorly to the currently available anti-epileptic drugs.

Adenosine is an inhibitory neuromodulator and has been proposed as an endogenous anticonvulsant molecule [73, 78]. An increase in extracellular adenosine and sustained enhancement in adenosine metabolites (including hypoxanthine, xanthine, and inosine) were observed in the brains of epileptic animal models, suggesting a compensatory protective mechanism in response to seizures [1, 111]. On the other hand, since A_1_R activation mediates presynaptic inhibition and stabilization of excitatory membrane potential, it has been logically speculated that dysregulation of A_1_R signaling may contribute to epileptogenesis. Pathological evidence has shown that A_1_R is downregulated in human temporal lobe epilepsy [92]. In the hippocampus of a kindled rat model, the density and neuromodulation effect of A_1_R were decreased [201]. Another study showed that A_1_R was unchanged at the protein level but was desensitized in status epileptic rats [101]. Despite downregulation and/or dysfunction, A_1_R activation has been widely shown to effectively suppress seizures in brain slices of patients with epilepsy [134] and epileptic animal models [83, 95, 101, 137]. These findings suggest that A_1_R activation may be a promising strategy for epilepsy management. Compared with A_1_R, less attention has been directed to the role of A_2A_R in epilepsy. Consistent with the neuronal facilitatory effect of A_2A_R signaling, the suppression of A_2A_R by genetic or pharmacological approaches has been reported to protect against seizures [75, 76]. The roles of A_2B_R and A_3_R in the pathogenesis of epilepsy are less evident.

Since A_1_R is a powerful treatment target for seizures, adenosine augmentation is considered an effective therapeutic strategy for epilepsy. Adenosine tone can be regulated by ENT1-mediated uptake machinery on the plasma membrane. The expression level of ENT1 is significantly increased in patients with epilepsy and in epileptic animal models [248, 259] (Table 1). Direct injection of a specific ENT1 inhibitor, nitrobenzylthioinosine, into the hippocampus ameliorated the severity of seizures and prolonged the onset latency of pilocarpine-induced seizures in rats [248]. Recently, systemic administration of BBB-permeable ENT1 inhibitors (JMF1907 and J4) produced beneficial effects in various seizure models that represent generalized tonic–clonic seizures, generalized myoclonic seizures, and focal seizure conditions [111] (Table 2). The anti-epileptic effects were associated with the suppression of excitatory neuronal activities by reducing the action potential-dependent release of neurotransmitters [111]. There was no direct evidence showing that specific adenosine receptors are involved in the protective effects of ENT1 inhibition in epilepsy.

Interestingly, a marijuana derivative (cannabidiol) has been shown experimentally and clinically to have anti-seizure properties for decades [36, 68, 69]. Given that cannabidiol is an ENT1 inhibitor [40], its anticonvulsant effects were proposed to be, at least in part, mediated by the increase in adenosine tone and the subsequent activation of A_1_R. Cannabidiol has been approved in the US and European Union for the treatment of seizures associated with Lennox-Gastaut syndrome, Dravet syndrome, or tuberous sclerosis complex [109, 216], supporting the idea of ENT1 inhibition for epileptic treatment, even for drug-resistant types. Nevertheless, in addition to the inhibition of ENT1, the molecular target(s) and effects of cannabidiol appear to be complex, including the activation of voltage-gated sodium channels, serotonin receptors, an orphan receptor (G protein-coupled receptor 55), nicotinic acetylcholine receptors, and anandamide transporters [97, 116].

In addition to ENT1, the role of ADK (a key adenosine-metabolizing enzyme) in the pathogenesis of epilepsy has been extensively explored in recent years. ADK is predominantly expressed in astrocytes and is believed to dictate intracellular adenosine levels. Astrogliosis is a pathological hallmark of epilepsy. In the human temporal lobe with epilepsy and in the hippocampus and cortex of rodent models with epilepsy, ADK was found to be overexpressed in reactive astrocytes [13, 165] (Table 1). The increased level of ADK was associated with overproduction of the proinflammatory cytokine interleukin-1β in epileptic conditions [13]. The critical role of ADK in epileptogenesis was confirmed by the findings that pharmacological and genetic inhibition of ADK suppressed seizure-like activities in epileptic models [96, 152] (Table 2). Interestingly, although A_1_R is involved in the protective effects of ADK inhibition under certain neuropathological conditions [161], it has been increasingly recognized that the anti-seizure effects of ADK inhibition are mainly mediated by the modulation of epigenetic machinery (e.g., DNA methylation) but not the adenosine receptor-dependent pathway. In the epileptic brain, the pathological increase in ADK levels was proposed to cause intracellular adenosine deficiency, sequentially driving the donation of a methyl group from SAM to DNA (Fig. 1). This modification shifts the brain into an aberrant hypermethylated state, in which the expression of many genes underlying neuronal excitation and epileptogenesis are dysregulated [136]. In agreement with this concept, in one study, adenosine treatment normalized DNA hypermethylation and prevented the progression of epilepsy development, regardless of whether a nonselective adenosine receptor antagonist (caffeine) was coadministered [241]. These data indicate that modulation of epigenetic modification by adenosine augmentation therapy has great potential for the treatment of epilepsy.

Both genetic and acquired epilepsies have been linked to the excessive activation of the mammalian target of rapamycin (mTOR) signaling pathway. Because mTOR signaling can be inhibited by AMPK activation-mediated phosphorylation of mTOR regulatory proteins, the role of AMPK in the development and intervention of epilepsy has attracted attention. In the brain tissues of humans with chronic seizures and animals with acute and chronic seizures, the protein expression of AMPK is reduced [252] (Table 1). In addition, AMPK-null mice were reported to have increased seizure susceptibility through unregulated mTOR signaling [179]. Consistently, pharmacological activation of AMPK by metformin, one of the most extensively prescribed antidiabetic drugs, attenuated seizure activity in a pilocarpine-induced epileptic model, with inhibited mTOR phosphorylation [172]. The anticonvulsant effects of metformin are also consistent with the suppression of overexpressed brain-derived neurotropic factor, a contributor to epileptogenesis [22, 172]. Moreover, chronic treatment with metformin decreased mortality and shortened the duration of generalized tonic–clonic seizures in an acute seizure model and reduced the duration of epileptic activity in a chronic seizure model [252]. These findings are summarized in Table 2. Notably, the AMPK-mTOR signaling pathway mediates a wide variety of cellular events. The detailed mechanism modulated by the AMPK-mTOR axis in epilepsy remains unclear and requires further investigation.

#### Neurodegenerative disease-associated sleep disorder

Sleep disturbances are commonly associated with neurodegenerative diseases and are known to greatly affect the quality of life of patients [119]. For example, sleep disorders, such as nighttime insomnia, daytime hypersomnia, and rapid eye movement (REM) sleep disorders, are often seen in patients with AD [159, 188] or PD [77, 253]. Many effective sedative-hypnotic drug products, including benzodiazepine and Z-drugs (e.g., zolpidem, zaleplon, zopiclone) are used to induce and/or maintain sleep in people with sleep disorders. Previous studies documented that the extracellular level of adenosine increases in the cortex and basal forebrain during wakefulness and decreases during sleep. The dynamic regulation of adenosine in the sleep–wake cycle suggests that adenosine is a homeostatic regulator of sleep [199]. Both A_1_R and A_2A_R have been implicated in sleep induction. Activation of A_2A_R is known to increase sleep, while the arousal effect of caffeine (a nonselective antagonist) is dependent on the blockade of A_2A_R [20, 115]. Consistent with these findings, oral administration of a BBB-permeable A_2A_R agonist (T1-11) increases nonrapid eye movement (NREM) sleep [123]. Since adenosine homeostasis is regulated by ENTs, the expression and functions of ENTs (especially ENT1) in the regulation of sleep have also been extensively investigated. For instance, sleep deprivation markedly reduces the amount of ENT1 protein (but not ENT1 transcript) in a specific brain region (the basal forebrain), suggesting that ENT1 is tightly regulated in sleep and that adenosine transport across the plasma membrane can be altered by sleep disruption [4]. In agreement with this concept, genetic removal of ENT1 impairs normal sleep–wake regulation [132]. Notably, sleep deprivation not only affects the level of ENT1 but also reduces the activity of ADK and 5’-endonucleotidases in the basal forebrain. Sleep disruption can thus alter cellular adenosine metabolism at multiple steps [3]. In contrast, the overexpression of ADK (the cytoplasmic isoform) markedly affects sleep physiology in mice and reduces NREM sleep enhanced by sleep deprivation [189]. It was therefore not surprising to find that the loss of CD73, a 5’-ectonuclotidase, increases the duration of NREM sleep and impairs the response of animals to sleep deprivation [260]. Collectively, these findings support the idea that adenosine homeostasis plays a central role in sleep regulation and that proteins involved in controlling adenosine metabolism are feasible drug targets for the development of new treatments for sleep disorders associated with neurodegenerative diseases.

### Potentials and challenges in the future development of adenosine-related therapies

Despite the tremendous efforts that have been devoted to studies of neurodegenerative diseases in the past two decades, effective treatments for many neurological and neurodegenerative diseases remain unmet medical needs. Since neurodegenerative diseases are characterized by specific protein pathologies (e.g., amyloid plaques and neurofibrillary tangles for AD, Lewy bodies for PD, and mHTT inclusions for HD), these proteins are considered as the primary driver of the corresponding disease. Unfortunately, despite promising preclinical success, most treatment attempts by directly targeting these neurotoxic proteins have failed to obtain satisfactory results in clinical studies. The potential difficulties causing such a gap between preclinical observations and clinical applications include inappropriate drug targets, animal models, intervention timing, and patient populations. Taking AD as an example, most advanced disease-modifying drugs under clinical development focus on β-amyloid or tau protein and therefore their treatment responses were usually demonstrated in animal models overexpressing these two targets, respectively. This is because no single animal model can fully recapitulate all the pathological phenotypes of human AD [17]. Before moving into clinical studies, careful evaluation of a therapeutic agent in additional animal models based on different mechanisms thus would be very helpful to reduce the risk of translational failure. Another concern is whether the treatment intervention is early enough to slow or halt neurodegeneration and behavioral declines. Most patients attending clinics usually have developed obvious clinical symptoms with significant protein pathology, neuronal loss, and even brain atrophy. It appears to be critical to initiate the treatment as early as possible. Most importantly, the complex mechanisms of human neurodegenerative diseases need to be extensively investigated in order to design effective therapeutic treatments. Notably, although neurodegenerative diseases are attributable to different neurotoxic proteins, most of them cause common early events, including dysregulations in energy metabolism, mitochondrial function, and neuroinflammation [100, 180]. At the early disease stage in which there may be no detectable clinical and functional abnormality, these pathogenic mechanisms, either alone or in combination, contribute to dysfunction of neurons and glial cells, thereby leading to harmful protein aggregation/accumulation and later neurotoxicity [55, 151]. The implications of adenosine homeostasis in the regulation of bioenergetic and inflammatory pathways as detailed in the previous section provide a strong rationale for exploring the benefit of adenosine-related therapies.

As detailed in this review, ample evidence suggests that adenosine-related mechanisms under various pathophysiological conditions may open up new avenues for the development of novel therapeutic approaches. In recent decades, targeting adenosine receptors has been extensively pursued, and the effort continues to evolve. Accumulating evidence suggests that activation of A_1_R and A_2A_R can be neuroprotective and neurodegenerative, respectively [74]. Thus, A_1_R agonists and A_2A_R antagonists have been developed as pharmacological agents. The challenges to developing adenosine receptor drugs have been discussed elsewhere [23, 74] and are not elaborated here. In brief, many orthosteric adenosine receptor agonists can cause adverse effects on cardiovascular systems. Although A_2A_R antagonists seem to be well tolerated, the effectiveness of A_2A_R ligands may depend on the treatment dosage, timing, and symptoms. For example, in a model of brain autoimmune disease, A_2A_R activation was shown to be beneficial at an early stage but detrimental at a later stage [117]. In addition, activation of A_2A_R improved motor function, whereas blockade of A_2A_R was beneficial for cognitive function in mouse models of HD [23].

In addition to agents targeting adenosine receptors, the development of pharmacological agents that modulate adenosine-metabolizing enzymes or transporters has attracted increasingly interest. Accumulating evidence has shown that these novel strategies (for example, ADK inhibitors and ENT inhibitors) have produced initial promising results in preclinical studies [23, 25, 46, 144]. Further optimization of these newly developed agents and the characterization of the underlying mechanisms warrant further investigation. Another interesting strategy for treating neurodegenerative diseases is the development of anti-inflammatory agents based on the control of adenosine homeostasis. This tactic is of great interest because adenosine, by acting through receptors, is recognized as an endogenous potent anti-inflammatory agent. Nonetheless, its role in the regulation of neuroinflammatory responses is relatively less clear. How neuroinflammation is modulated in the brain by adenosine-related mechanisms is discussed below.

#### Challenges in the development of ADK inhibitors

Adenosine augmentation therapy has been shown to be beneficial for several brain disorders, particularly epilepsy. Proof-of-concept studies of several ADK inhibitors have been carried out successfully in animal models of epilepsy [139, 170]. Nonetheless, further development of these inhibitors is hindered by adverse reactions (including cardiovascular events and hepatic steatosis) observed during chronic application [26, 96, 239]. It has been proposed that these limitations can be overcome by two strategies. First, using ADK inhibitors in a short-term regimen would help to reduce systemic toxicities. A novel treatment methodology has been successfully developed in which the transient use of a small-molecule ADK inhibitor during the critical stage of epileptogenesis provides disease-modifying effects against epilepsy in animals [206]. Second, the development of selective inhibitors for the nuclear isoform of ADK would reduce the treatment dose of traditional nonselective ADK inhibitors. There are two isoforms of ADK, namely, ADK-long (ADK-L) and ADK-short (ADK-S), in mammalian cells. ADK-L and ADK-S were shown to be localized in the nucleus and cytoplasm, respectively [62], suggesting a specific function for the long isoform in gene regulation. Consistently, ADK-L is significantly more effective than ADK-S in the regulation of DNA methylation [241]. These observations, combined with the finding that ADK-L is predominantly expressed in the brain [61], suggest that targeting ADK-L might lead to the development of a more efficient way of modulating epigenetics in epileptogenesis. Further studies are needed to test whether novel approaches based on ADK inhibition can satisfy the requirements for efficacy and safety.

#### Challenges in the development of ENT inhibitors

ENTs play essential roles in the control of adenosine homeostasis. The function of ENT1 in the transport of adenosine, regulating cellular functions in the brain, has been extensively studied [214]. As described above, several BBB-permeable ENT1 inhibitors have recently been developed as potential drug candidates for the treatment of multiple neurodegenerative diseases, including AD, HD, ALS, and epilepsy [46, 111, 127, 141, 144]. Although the safety of systemic inhibition of ENT1 using these novel compounds has not yet been revealed, it is unlikely to be a major concern because several potent, but BBB impermeable, ENT1 inhibitors (such as ticagrelor and dipyridamole) have been widely used in the clinic [29, 42]. For future development, the safety profiles of these ENT1 inhibitors in the CNS need to be carefully evaluated before they are entered into clinical trials.

The roles of another ENT member, ENT2, in healthy and diseased brains have been recently investigated. They are very important for the control of adenosine augmentation because the transcript level of ENT2 is significantly higher than that of ENT1 in the brain. In addition, using a genetic deletion approach, ENT2 was shown to be the key determinant for modulating adenosine levels in the brain, particularly in the hippocampus [244]. Because ENT2 exhibits lower affinity and higher capacity than ENT1, it has been postulated that ENT2 is the dominant transporter when the adenosine level is dramatically elevated under certain pathological conditions. In line with this hypothesis, the extracellular adenosine level was found to be significantly elevated in ENT2-knockout mice, especially under LPS-challenge conditions, thereby protecting against LPS-induced neuroinflammation, BBB damage, and memory impairment [243, 244]. Given that neuroinflammation is critical for the pathogenesis of almost all neurodegenerative diseases, ENT2 inhibition may be a potential therapeutic strategy for these disorders in the brain. Unfortunately, to the best of our knowledge, no ENT2-specific inhibitor has been developed thus far. At this time, only a few compounds (e.g., soluflazine, R70960) that have a relatively higher affinity for ENT2 than for ENT1 have been reported [102, 229]. To target brain disorders associated with neuroinflammation, the development of novel ENT2-specific inhibitors that are BBB permeable is highly desirable. Given that no obvious defect in global ENT2-knockout mice was observed [243, 244], systemic inhibition of ENT2 may be a feasible pharmacological approach.

#### Modulation of neuroinflammation by adenosine-related mechanisms

Neuroinflammation is a common feature of neurological and neurodegenerative diseases. Adenosine has been recognized as an anti-inflammatory molecule because the augmentation of endogenous adenosine diminishes inflammation in peripheral systems [2, 121, 178]. In the CNS, direct adenosine treatment and the activation of several adenosine receptors (e.g., A_2A_R, A_2B_R, and A_3_R) attenuated the LPS-induced upregulation of proinflammatory cytokines and increased the production of anti-inflammatory cytokines in microglia [138, 148, 223]. In addition, adenosine augmentation, induced by either the inhibition of ENTs or ADK or the activation of A_1_R or A_2A_R, alleviated neuroinflammation in some pathological animal models of AD and PD and neuroinflammation induced by intracerebral hemorrhage, multiple sclerosis, or LPS [46, 114, 168, 220, 244]. However, opposite results showed that A_2A_R signaling is involved in the facilitation of neuroinflammation and is associated with neuronal injuries [38, 175, 202]. These seemingly controversial data indicate that adenosine is undoubtedly an immunomodulator in the brain, but the outcome of adenosine receptor-mediated signaling may be dependent on the disease type, disease stage, and brain area involved. For example, it was reported that A_2A_R activation exhibited anti-inflammatory effects and prevented pathological symptoms only when the treatment was introduced at an early stage of autoimmune neuroinflammation in the brain. In contrast, A_2A_R activation in the late stage was detrimental [117]. An additional issue is the affected area(s). The differential distribution and interplay of adenosine receptors among themselves and/or with other types of receptors may lead to region-specific regulation of neuroinflammatory responses. Moreover, under certain pathological conditions, compromised BBB and aberrant interaction between the central and peripheral immune systems may contribute to neuroinflammation [66, 128]. Therefore, the combined impact of adenosine modulation on central and peripheral immune responses may be considered. Furthermore, other coexisting factors may modulate the role of adenosine receptors on immune responses. For example, the local level of glutamate, which may be dramatically increased during brain injury, switches the effect of A_2A_R activation from a proinflammatory to an anti-inflammatory status [65]. Intriguingly, increased adenosine tone and A_1_R activation in ENT2-knockout mice under neuroinflammatory conditions normalized excessive extracellular glutamate by increasing the level of a major glutamate reuptake transporter, EAAT2 [243]. In this circumstance, activation of both A_1_R and A_2A_R may have led to anti-inflammatory effects [244]. It remains unknown whether ENT2 inhibition produces any effect on chronic inflammation in the context of neurodegenerative diseases. Further investigations are required to advance our current understanding regarding how adenosine modulates neuroinflammation caused by different insults, and the findings may offer new insights into the development of novel therapeutic treatments for neurodegenerative diseases.

## Conclusions

Ample evidence suggests that the tight control of adenosine levels, a measure of adenosine homeostasis, is critical for the maintenance of cellular functions, particularly energy production and gene regulation [46, 122, 171, 192, 207, 238]. This regulatory importance may explain why coordinated regulation of adenosine metabolic enzymes under various pathophysiological conditions has been reported and may support the contention that adenosine homeostasis undergoes dynamic regulation induced by environmental cues. Moreover, adenosine homeostasis not only plays a critical role in the regulation of neuronal functions/plasticity but also greatly contributes to the interaction between neurons and glia [6, 59, 105, 106]. Assessing the interaction between brain cells in vivo is a challenging task. The recent development of single-cell transcriptome profiling technology is expected to greatly facilitate the delineation of adenosine homeostasis regulation among different types of brain cells (e.g., neurons vs. glia), especially in neuroinflammation. Notably, isotype-specific antibodies and pharmacological tools (i.e., activators, allosteric modulators, and inhibitors) are definitely required for in-depth investigations and future clinical applications. Some of these tools are currently unavailable due to the lack of comprehensive knowledge regarding the structure and function of the proteins that govern adenosine metabolism. Specifically, antibodies and isotype-specific inhibitors of the four members of the ENT family are not commonly available at this time, thus hindering the understanding of the functional roles of these ENTs in neurodegenerative diseases. As an increasing number of high-resolution protein structures have been resolved by cryo-electron microscopy, the protein structures of ENTs may also be thus obtained to facilitate the development of specific antibodies and/or inhibitors for ENTs. These new tools together with the intriguing findings on dysregulated adenosine homeostasis in neurodegenerative diseases are the bases for further investigation, which may pave the wave for the development of novel therapeutic treatments in the future.

## Data Availability

Not applicable.

## Abbreviations

5’UTR: 5’ Untranslated region
6-OHDA: 6-Hydroxydopamine
Aβ: β-Amyloid
AC: Adenylyl cyclase
AD: Alzheimer's disease
ADA: Adenosine deaminase
ADK: Adenosine kinase
ADP: Adenosine diphosphate
AK: Adenylate kinase
ALS: Amyotrophic lateral sclerosis
AMP: Adenosine monophosphate
AMPA: α-Amino-3-hydroxy-5-methyl-4-isoxazolepropionic acid
AMPK: AMP-activated protein kinase
ATP: Adenosine triphosphate
BBB: Blood–brain barrier
CNS: Central nervous system
CNT: Concentrative nucleoside transporters
CREB: CAMP-response element binding protein
CSDS: Chronic social defeat stress
D2R: Dopamine receptor 2
DNA: Deoxyribonucleic acid
DNMTs: DNA methyltransferases
EAAT2: Excitatory amino acid transporter 2
ENT: Equilibrative nucleoside transporters
GABA: γ-Aminobutyric acid
GPCR: G protein-coupled receptor
HD: Huntington’s disease
iPSC: Induced pluripotent stem cell
LPS: Lipopolysaccharide
mHTT: Mutant Huntingtin
MFF: Mitochondrial fission factor
MPTP: 1-Methyl-4-phenyl-1,2,3,6-tetrahydropyridine
MSN: Medium spiny neuron
mTOR: Mammalian target of rapamycin
NDPK: Nucleotide diphosphokinase
NLRP3: NLR family pyrin domain containing 3
NREM: Non-rapid eye movement
P2Y12: Purinergic receptor P2Y, G-protein coupled,12
PEXN motif: Amino acid residues Pro71, Glu72, and Asn74
PD: Parkinson's disease
PKA: Protein kinase A
PolyQ: Polyglutamine
REM: Rapid eye movement
SAH: S-adenosylhomocysteine
SAHH: S-adenosylhomocysteine hydrolase
SAM: S-adenosylmethionine
TDP-43: TAR DNA binding protein 43
ULK1: Unc-51 Like Autophagy Activating Kinase 1
